# The effect of short-duration sub-maximal cycling on balance in single-limb stance in patients with anterior cruciate ligament injury: a cross-sectional study

**DOI:** 10.1186/1471-2474-5-44

**Published:** 2004-11-17

**Authors:** Eva Ageberg, David Roberts, Eva Holmström, Thomas Fridén

**Affiliations:** 1 Department of Rehabilitation, Lund University Hospital, Lasarettsgatan 13, SE-221 85 Lund, Sweden; 2 Department of Orthopedics, Lund University Hospital, SE-221 85 Lund, Sweden; 3 Department of Physical Therapy, Lund University, Lasarettsgatan 7, SE-221 85 Lund, Sweden

## Abstract

**Background:**

It has previously been shown that an anterior cruciate ligament (ACL) injury may lead to impaired postural control, and that the ability to maintain postural control is decreased by fatigue in healthy subjects. To our knowledge, no studies have reported the effect of fatigue on postural control in subjects with ACL injury. This study was aimed at examining the effect of fatigue on balance in single-limb stance in subjects with ACL injury, and to compare the effects, and the ability to maintain balance, with that of a control group of uninjured subjects.

**Methods:**

Thirty-six patients with unilateral, non-operated, non-acute ACL injury, and 24 uninjured subjects were examined with stabilometry before (pre-exercise) and immediately after (post-exercise) short-duration, sub-maximal cycling. In addition, the post-exercise measurements were compared, to evaluate the instantaneous ability to maintain balance and any possible recovery. The amplitude and average speed of center of pressure movements were registered in the frontal and sagittal planes. The paired t-test was used for the intra-group comparisons, and the independent t-test for the inter-group comparisons, with Bonferroni correction for multiple comparisons.

**Results:**

No differences were found in the effects of exercise between the patients and the controls. Analysis of the post-exercise measurements revealed greater effects or a tendency towards greater effects on the injured leg than in the control group. The average speed was lower among the patients than in the control group.

**Conclusions:**

The results of the present study showed no differences in the effects of exercise between the patients and the controls. However, the patients seemed to react differently regarding ability to maintain balance in single-limb stance directly after exercise than the control group. The lower average speed among the patients may be an expression of different neuromuscular adaptive strategies than in uninjured subjects.

## Background

The anterior cruciate ligament (ACL) is the most commonly injured ligament in the knee. The risk of future joint problems, in the form of functional limitations, secondary lesions, and arthrosis, is increased following such an injury. Secondary effects commonly seen after an ACL injury include defective neuromuscular function with reduced strength and functional performance, a different movement and activation pattern, defective proprioception and impaired postural control [1]. Impaired postural control has been reported after acute [2], and chronic ACL injury [3-5], as well as after ACL reconstruction [6-8]. Higher amplitude values [2-5] and longer reaction time when subjected to perturbations [4,6,7] have been observed among patients compared to controls. Studies have also shown that patients with better subjective function have lower amplitude values [[8], Ageberg E, Roberts D, Holmström E, Fridén T: Balance in single-limb stance in individuals with anterior cruciate ligament injury – relation to knee laxity, proprioception, muscle strength, and subjective function. Manuscript submitted]. The present study was initiated by the clinical knowledge that although patients with ACL injury have had extensive neuromuscular training and function well during daily life and (modified) physical activities, they experience a decreased ability to maintain balance during weight-bearing on the injured leg in demanding situations while fatigued. This may be related to an increased risk of further injuries.

Fatigue is caused by a combination of different physiological mechanisms occurring at both the central and peripheral levels [9], affecting afferent neuromuscular pathways, observed as proprioceptive deficiency [10-12], and efferent neuromuscular pathways, seen, for example, as a delay in muscle response [13,14]. Thus, muscular fatigue leads to a decline in work performance, which may also include effects on postural control. A decreased ability to maintain balance in bilateral stance [15-17], and single-limb stance [15,18-20] after fatiguing exercise (i.e., higher values after exercise) has been reported in uninjured subjects, and it has been suggested that individuals are therefore at increased risk of injury when fatigued [15,19,20]. Studies of balance in single-limb stance are of importance and of interest since these movement patterns resemble the stance phase, and since many knee injuries occur during weight-bearing on one leg [21].

To our knowledge, no studies evaluating the effect of fatigue on postural control in subjects with ACL injury have been reported. The main purposes of this study were: 1) to examine the effect of short-duration, sub-maximal exercise performed on a cycle ergometer, on postural control, measured by stabilometry in single-limb stance on a force platform, in individuals with ACL injury in comparison with that of a control group of uninjured subjects, and 2) to explore the patients' instantaneous ability to maintain balance in single-limb stance after exercise, in comparison with that of the control group. Furthermore, the patients were compared to the control group in order to verify previous findings that postural control is affected in both legs by a unilateral ACL injury [2-5]. No comparisons were, therefore, made between the injured and uninjured legs. Our hypothesis was that the patients with ACL injury would be more affected by exercise than the uninjured subjects, since fatigue has been shown to reduce postural control in healthy subjects, and since postural control may already be impaired due to the injury.

## Methods

### Patients

Thirty-six patients (18 men and 18 women) were included in the study. Inclusion criteria were: 1) age between 15 and 35 years, 2) unilateral, non-operated, non-acute ACL deficiency with or without associated lesions of other structures of the knee, 3) an uninjured contralateral extremity, back and neck, and 4) no history of neurological disease, vestibular or visual disturbance. Their mean age was 26 years (SD 5 years), mean height 174 cm (SD 9 cm), and mean body mass 72 kg (SD 13 kg). Their median activity level before injury was 6.5 (range 3 to 9) and on the test occasion 4 (range 1 to 9) according to the Tegner activity level scale [22]. The mean time elapsed from injury to the test occasion was 3.8 years (SD 3, range 0.5 to 11 years). The patients had undergone an extensive neuromuscular training program [23] after the injury under the supervision of physical therapists, with a mean duration of 7 months (SD 5 months). A visual analog scale graded from 0 to 100 mm was used for subjective evaluation of extremity function, where 0 was "as if the knee had been recently injured" and 100 was "perfect" [24]. The patients' mean value and median value on this scale were 68 mm and 59 mm (range 12–95 mm), respectively.

### Control group

The measurements of twenty-four uninjured volunteers (11 men and 13 women) from a previous study [18], with no history of neurological disease, major orthopedic lesion, vestibular or visual disturbance, constituted control values. Their mean age was 24 years (SD 3 years), mean height 176 cm (SD 8 cm), and mean body mass 71 kg (SD 13 kg). Their median activity level was 5 (range 2 to 9) according to the Tegner activity level scale [22]. The subjects in the control group were chosen in order to have the same distribution in age, sex, and physical activity as the patients [25]. No significant difference was found between the groups in age, height, body mass or activity level. The Research Ethics Committee at Lund University approved the study. All subjects gave their written informed consent to participate in the study.

### Assessment

#### Stabilometry

**Figure 1:**
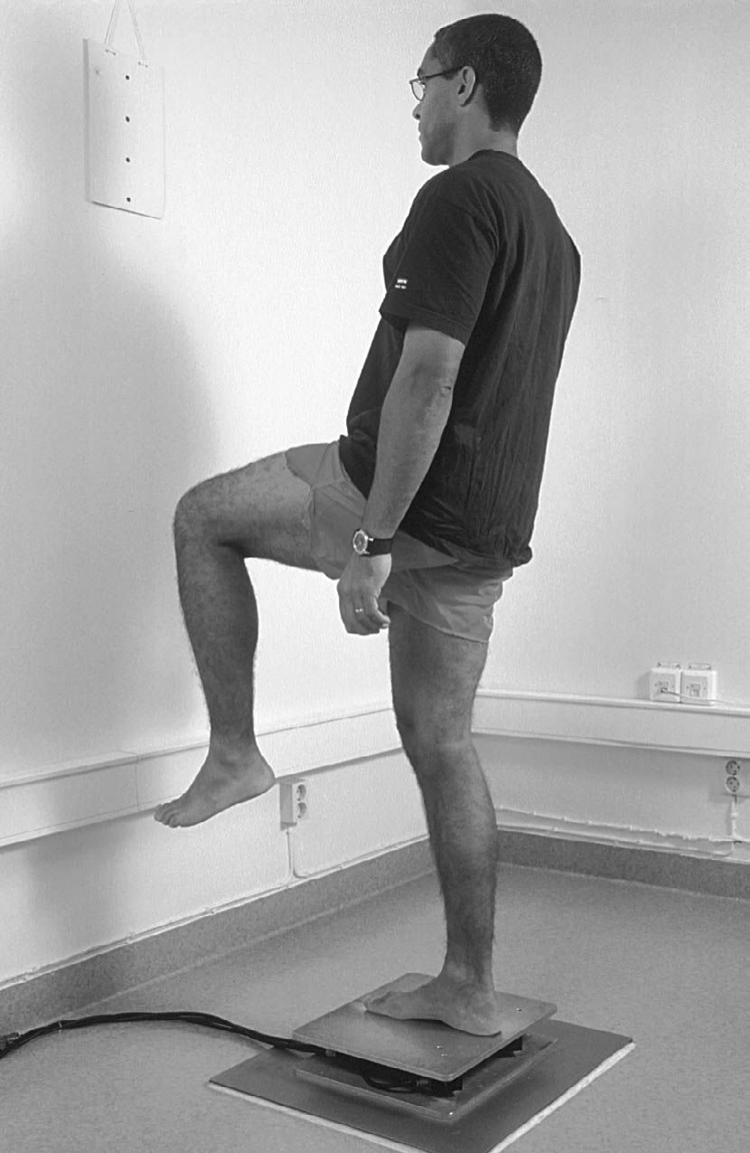
**Stabilometry in single-limb stance, tested by means of a strain gauge force plate**. A standardized position was used. The subject is a model who did not participate in the study. Reprinted from Ageberg E, Zätterström R, Moritz U, Fridén T. Influence of supervised and nonsupervised training on postural control after an acute anterior cruciate ligament rupture: A 3-year longitudinal prospective study. *Journal of Orthopaedic & Sports Physical Therapy*. 2001:31: 632–644, with permission of the Orthopaedic and Sports Sections of the American Physical Therapy Association.

**Figure 2:**
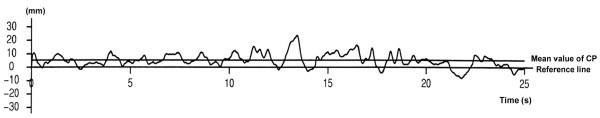
**Raw data from a stabilometry trial**. A measurement in the frontal plane for the right leg in a subject who did not participate in the study. The reference line, the mean value of center of pressure (CP), and movements of the CP are shown in the Figure. In this trial, the average speed was 20 mm·s^-1 ^and the number of DEV 10 was 5.

Balance in single-limb stance was tested by means of a strain gauge force plate (33 × 38 cm) with the subject barefoot in a standardized position [5,26,27] (Figure 1). This measurement was performed before (pre-exercise) and immediately after fatiguing exercise (post-exercise). The foot was placed pointing straight forward in relation to reference lines in the frontal and sagittal planes (origin of coordinates). The other leg was flexed 90° at the hip and knee joints with both arms hanging relaxed at the sides. The subjects were instructed to stand as motionless as possible, looking straight ahead at a point on the wall 65 cm away; they were allowed to practice maintaining this position for about 20 s before three measurements on each leg were made, with the subjects standing alternately on their right and left leg. The test order between legs was randomized regarding injured/uninjured leg in the patient group (injured leg n = 20, uninjured n = 16), and regarding right/left leg in the control group (right leg n = 13, left n = 11). No differences were observed in the stabilometric measurements between these randomization groups. Accordingly, the assessment included three measurements made on each leg, giving a total of six measurements pre- and post-exercise, respectively. These six measurements lasted for approximately 3 1/2 minutes, with about 10 seconds between each measure. The median value of the three measurements on each leg was used to compare pre- and post-exercise values. Decreasing values in the three measurements have been observed in a previous study, indicating a learning effect [26]. Some degree of recovery may, therefore, occur during the three post-exercise measurements. For this reason, the first and third of the three post-exercise measurements on each leg were used, to evaluate the instantaneous value of the ability to maintain postural control (first measurement) and the possible recovery (third measurement). Movements of the center of pressure (CP) in the frontal plane (FP) and sagittal plane (SP) were recorded for 25 s at a sampling frequency of 20 Hz. A computer program (Viewdac 2.1, Keithley Instruments, Inc., Cleveland, Ohio, USA), was used to analyze the following variables: *1) average speed of CP movements in mm·s^-1^; and 2) number of movements exceeding 10 mm from the mean value of CP (DEV 10)*, giving a total of four variables (two variables in each plane). The mean value of CP is the average distance (mm) of the CP from the reference lines, and DEV 10 is the number of movements exceeding 10 mm from the mean value of CP. DEV 10 (n) reflects the deviation of CP (i.e., displacement of CP), and the average speed (mm·s^-1^) reflects the amplitude and frequency of CP movements. Figure 2 shows raw data from a stabilometry test. Average speed and DEV 10 were used in the present study, since our previous studies have shown that these variables are reliable [18,26], and sensitive in detecting differences between patients and uninjured subjects [2], and sensitive in detecting the effects of exercise [18]. We expected to find higher values after exercise [18].

#### Short-duration sub-maximal exercise

Short-duration, sub-maximal exercise was performed on a cycle ergometer. The subjects' heart rate was continuously recorded during the entire test. Borg's scale for Rating of Perceived Exertion (RPE scale) was used to assess the subjective effort level during exercise [28]. On this scale, numbers ranging from 6 to 20 are matched with descriptors (e.g., 6 = *No exertion at all*, 13 = *Somewhat hard*, 15 = *Hard*, 17 = *Very hard*, 19 = *Extremely hard*, and 20 = *Maximal exertion*). The RPE scale was designed to increase linearly with exercise intensity and heart rate for work on a bicycle ergometer, and correlates closely with several physiological variables, including heart rate and blood lactate concentration [28]. A linear relationship exists between heart rate and oxygen consumption with increasing rate of work. A given percentage of the maximum oxygen consumption (VO_2max_) results in a higher percentage of the maximum heart rate (HR_max_); e.g., 75% of VO_2max _represents an intensity of 86% of HR_max _[29]. The maximum heart rate can be estimated from the following equation: maximum heart rate (beats/min) = 220 – age (years) [29]. Effects of fatigue are likely to occur after a few minutes of sub-maximal exercise [9].

The rate of pedaling was kept constant at 60 revs/min. The level of exercise was calculated so as to be similar to that perceived during a general exercise session. The workload (W) was set individually, depending on the sex and physical condition of each subject, with the aim of reaching a heart rate above 60% of the predicted HR_max _[30] in all subjects. Cycling was stopped when the subjects had reached a heart rate exceeding 60% of the predicted HR_max_, perceived the exercise as hard or very hard (values 14–17 of the RPE scale), and had reached steady-state heart rate, i.e., after approximately 5 min.

### Statistical analysis

The average of the right and left legs; i.e., (right+left)/2, was used for statistical analysis in the control group, since there were no clinically or statistically significant differences between the legs. The use of the mean value of both legs when performing parametric statistics can be questioned, since this may affect the data variability. It cannot, however, be excluded that a dominance of one or the other side exists, which is difficult to define [25], and therefore it is hard to determine which leg to use in comparison with the patients. For this reason we used the average of the right and left legs. However, the results were confirmed using the right and left legs separately as the control leg. The median value of the three measurements was used to compare pre- and post-exercise values. In addition, the first and third of the three post-exercise measurements were compared, to evaluate the instantaneous ability to maintain postural control in single-limb stance (first measurement) and the possible recovery (third measurement). We used the paired t-test for the intra-group comparisons, and the independent t-test for the inter-group comparisons, with Bonferroni correction for multiple comparisons. The present study is of exploratory character, and the level of correction for multiple comparisons was chosen with regard to this. For each stabilometric variable, five separate t-tests were performed in comparisons between pre- and post-exercise values for the injured leg and the control group: 1) injured leg pre-exercise vs. post-exercise, 2) control group pre-exercise vs. post-exercise, 3) injured leg vs. control group pre-exercise, 4) injured leg vs. control group post-exercise, and 5) effects of exercise (post-exercise minus pre-exercise) injured leg vs. control group. These five t-test were also performed in the analysis of possible differences between pre- and post-exercise values for the uninjured leg and the control group. Since five comparisons were made in the above-mentioned analyses, the alpha level was set at 0.05/5 = 0.01. For each stabilometric variable, three separate t-tests were performed in comparisons between post-exercise measurements 1 and 3 for the injured leg and the control: 1) injured leg measurement 1 vs. measurement 3, 2) control group measurement 1 vs. measurement 3, and 3) effects of exercise (measurement 3 minus measurement 1) injured leg vs. control group. These three t-tests were also performed in the analysis of possible differences between post-exercise measurements 1 and 3 for the uninjured leg and the control group. Since three comparisons were made in the above-mentioned analyses, the alpha level was set at 0.05/3 = 0.02. The statistical analyses were performed using the program package SPSS 11.0 (SPSS Inc., Chicago, Illinois, USA).

## Results

### Fatiguing exercise

All subjects exceeded the 60% value of the predicted HR_max_; the mean level being 82% (SD 6%, range 66 to 92%) among the patients and 81% (SD 7%, range 68 to 99%) among the controls. The median power output produced by the patients and the control group at the end of fatiguing exercise was 125 W (range 75 to 200 W) and 150 W (range 100 to 200 W), respectively, and the mean value of perceived exertion, rated according to the RPE scale, was 15.8 (SD 1.1) and 15.4 (SD 0.9), respectively. The final heart rate attained among the patients and the control group was 159 beats/min (SD 11 beats/min) and 159 beats/min (14 beats/min), respectively, and the heart rate after the stabilometric assessment, approximately 3 1/2 minutes after exercise, was 112 beats/min (SD 14 beats/min) and 117 beats/min (SD 16 beats/min), respectively. No significant differences were found between the patients and controls with regard to the above-mentioned variables.

### Average speed of CP movements

**Figure 3:**
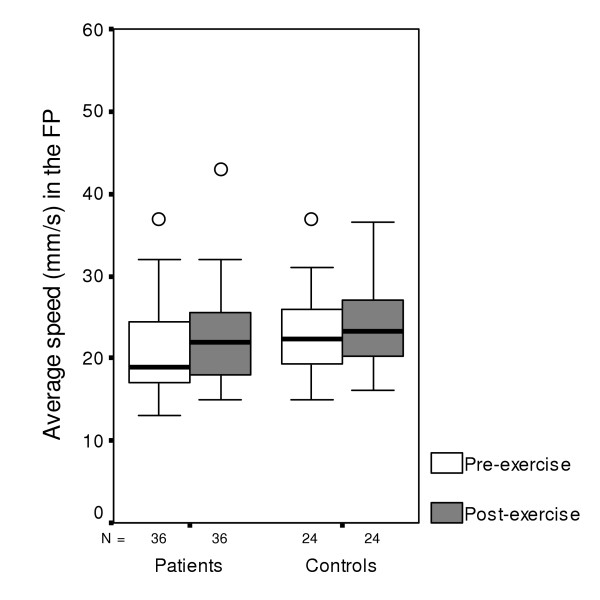
**Pre- and post-exercise values**. Average speed (mm·s^-1^) in the frontal plane (FP) pre- and post-exercise, for the injured leg of the patients and the uninjured legs of the control group. The circles denote outliers (i.e., cases with values between 1.5 and 3 box lengths from the upper or lower edge of the box).

**Figure 4:**
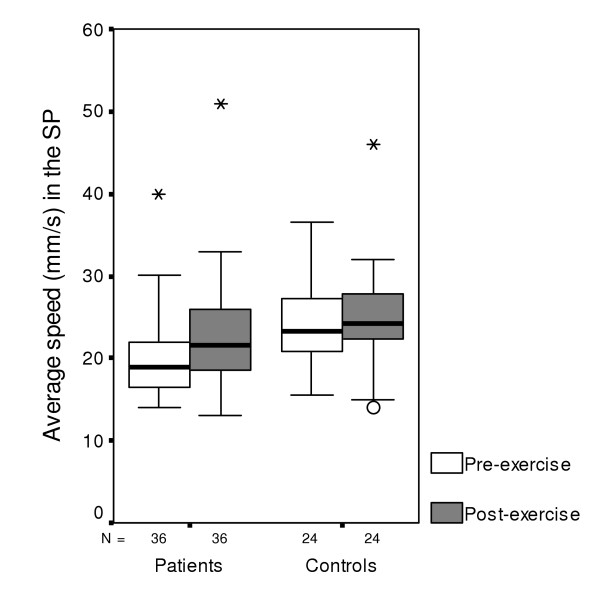
**Pre- and post-exercise values**. Average speed (mm·s^-1^) in the sagittal plane (SP) pre- and post-exercise, for the injured leg of the patients and the uninjured legs of the control group. The asterisks denote extreme values (i.e., cases with values more than 3 box lengths from the upper or lower edge of the box).

Higher values were noted post- than pre-exercise in the FP and SP in the injured and uninjured legs, but only in the FP in the control group (Table 1). No differences were noted between the groups regarding the effects of exercise (mean difference of post-exercise minus pre-exercise values) (Table 2). Figures 3 and 4 show the pre-and post-exercise values for the injured leg and the control group.

A lower value was observed in the third than in the first of the post-exercise measurements on the injured leg in the FP, but no differences were noted on the uninjured leg or in the control group (Table 3). The injured leg of the patients was more affected by exercise directly after cycling than the legs of the control group in the FP (Table 4). Figures 7 and 8 show the first and third of the post-exercise measurements on the injured leg and in the control group.

Lower values were observed pre-exercise in the SP in the injured and uninjured legs of the patients than in the control group (Table 5).

### Number of movements exceeding 10 mm from the mean value of CP

**Figure 5:**
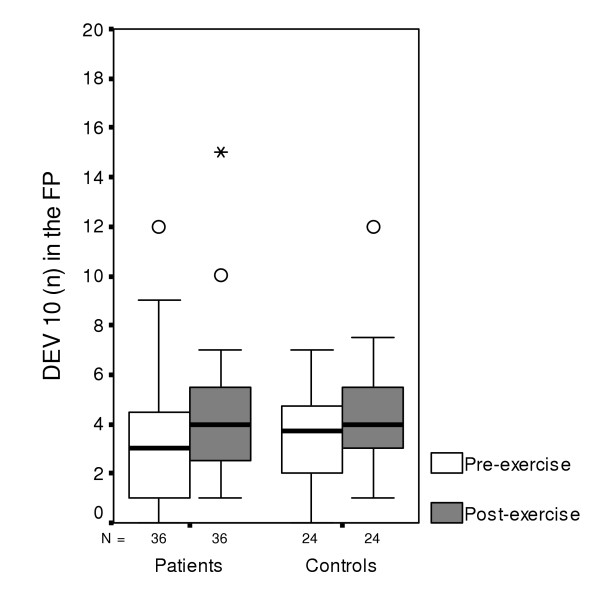
**Pre- and post-exercise values**. DEV 10 (n) in the frontal plane (FP) pre- and post-exercise, for the injured leg of the patients and the uninjured legs of the control group. The circles denote outliers (i.e., cases with values between 1.5 and 3 box lengths from the upper or lower edge of the box), and the asterisks denote extreme values (i.e., cases with values more than 3 box lengths from the upper or lower edge of the box).

**Figure 6:**
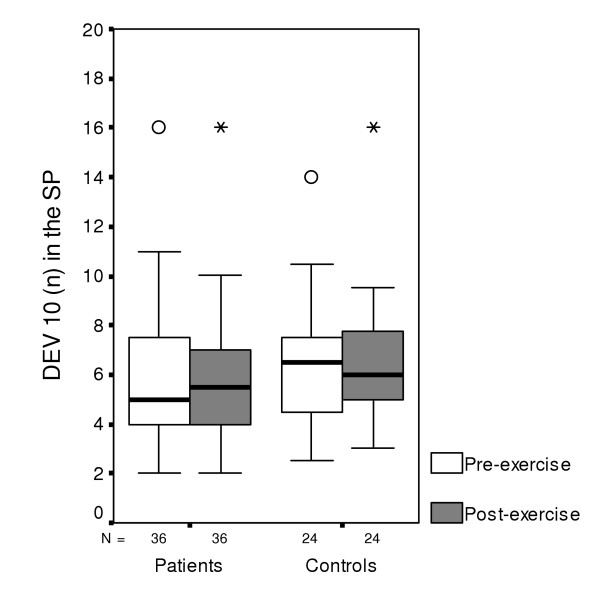
**Pre- and post-exercise values**. DEV 10 (n) in the sagittal plane (SP) pre- and post-exercise, for the injured leg of the patients and the uninjured legs of the control group. The circles denote outliers (i.e., cases with values between 1.5 and 3 box lengths from the upper or lower edge of the box), and the asterisks denote extreme values (i.e., cases with values more than 3 box lengths from the upper or lower edge of the box).

A higher value was found post- than pre-exercise in the uninjured leg in the FP, and the post-exercise value tended to be higher in the injured leg and in the control group (Table 1). No differences were found between pre- and post-exercise values in the SP (Table 1), or between the groups regarding the effects of exercise (mean difference of post-exercise minus pre-exercise values) (Table 2). Figures 5 and 6 show the pre-and post-exercise values for the injured leg and the control group.

The third of the post-exercise measurements was lower than the first in the injured leg in both planes, but no differences were found for the uninjured leg or in the control group (Table 3). No differences were noted between the groups regarding the effects of exercise directly after cycling (Table 4). Figures 9 and 10 show the first and third of the post-exercise measurements on the injured leg and in the control group.

No differences were found between the injured leg and the control group, or between the uninjured leg and the control group (Table 5).

## Discussion

Short-duration, sub-maximal exercise on a cycle ergometer resulted in increased average speed in both planes, and in the amplitude of CP movements (DEV 10) in the FP during balance in single-limb stance among the patients with ACL injury. In the intra-group comparisons, three of four variables showed higher values post- than pre-exercise in the uninjured leg, and two of four variables were higher post-exercise in the injured leg. In the control group, one of four variables was higher post- than pre-exercise (Table 1). However, no differences in the effects of fatigue (mean difference of post-exercise minus pre-exercise values) were found in the inter-group comparisons (Table 2, and Figures 3, 4, 5, 6). The variables were more sensitive in detecting the effects of exercise in the FP than in the SP. The primary motions of the knee joint occur in the SP, and the joint has limited capacity to make postural adjustments in the FP due to anatomical constraints, whereas the hip joint and ankle are involved in postural corrections in both the FP and SP during weight-bearing [31]. Since many injuries to the knee occur during weight-bearing on one leg [21]; i.e., in a closed kinetic chain including the hip joint and ankle, it is of interest to examine postural control in both the FP and SP in individuals with ACL injury. The results of a previous study [18] and the present one indicate that measurements in the FP may be more sensitive and revealing in detecting effects of exercise than measurements in the SP.

It has been demonstrated that afferent information has an effect on the neuromuscular function of both the ipsilateral and contralateral limb muscles [32], which may explain why more variables were higher post- than pre-exercise not only in the injured leg, but also in the uninjured one, than in the control group. Several studies have reported bilateral defects in postural control after an ACL injury or reconstruction [2-7], which may be due to central nervous system modifications following the loss of knee mechanoreceptors after the injury [33,34]. Another explanation may be that the patients had inherently poor balance, which might have contributed to the original injury. This has been reported by Tropp et al. [35], where soccer players with abnormal stabilometric values (defined as a value exceeding 2 SD of the mean value in a control group), ran a higher risk of sustaining an ankle injury than players with normal values.

**Figure 7:**
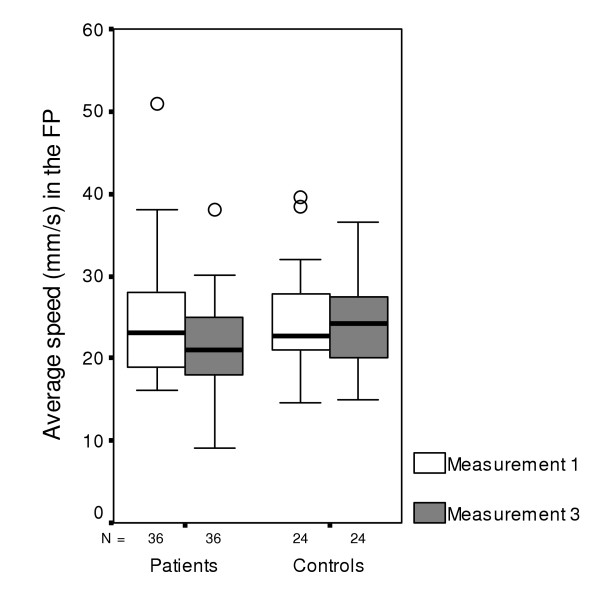
**Post-exercise measurements 1 and 3**. Average speed (mm·s^-1^) in the frontal plane (FP) post-exercise measurements 1 and 3, for the injured leg of the patients and the uninjured legs of the control group. The circles denote outliers (i.e., cases with values between 1.5 and 3 box lengths from the upper or lower edge of the box).

**Figure 8:**
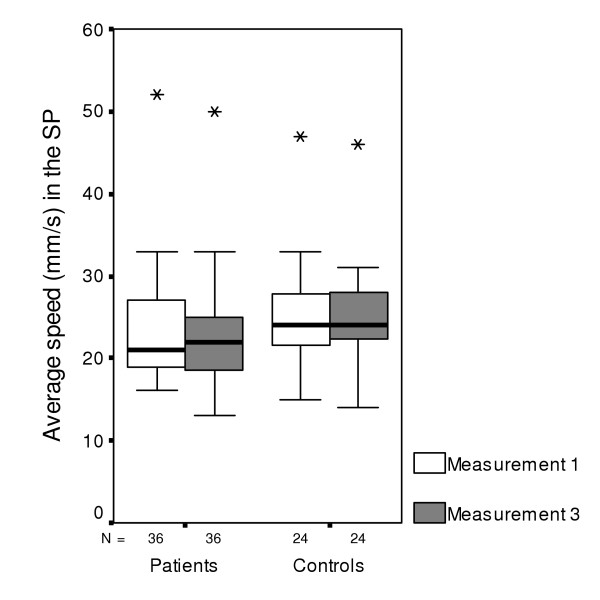
**Post-exercise measurements 1 and 3**. Average speed (mm·s^-1^) in the sagittal plane (SP) post-exercise measurements 1 and 3, for the injured leg of the patients and the uninjured legs of the control group. The asterisks denote extreme values (i.e., cases with values more than 3 box lengths from the upper or lower edge of the box).

**Figure 9:**
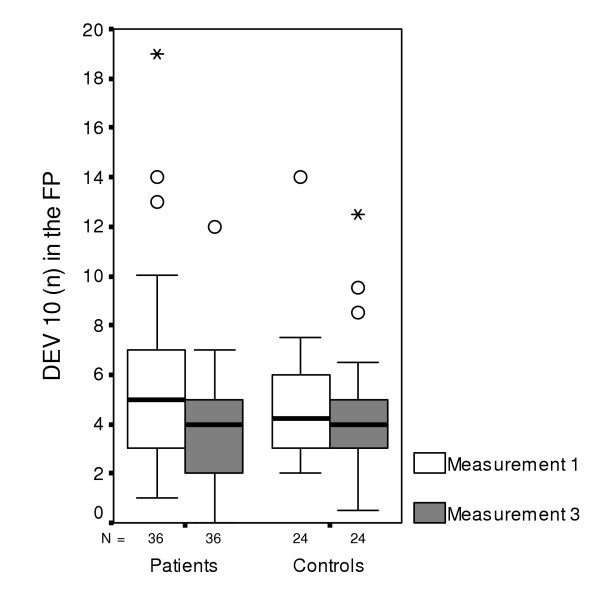
**Post-exercise measurements 1 and 3**. DEV 10 (n) in the frontal plane (FP) post-exercise measurements 1 and 3, for the injured leg of the patients and the uninjured legs of the control group. The circles denote outliers (i.e., cases with values between 1.5 and 3 box lengths from the upper or lower edge of the box), and the asterisks denote extreme values (i.e., cases with values more than 3 box lengths from the upper or lower edge of the box).

**Figure 10:**
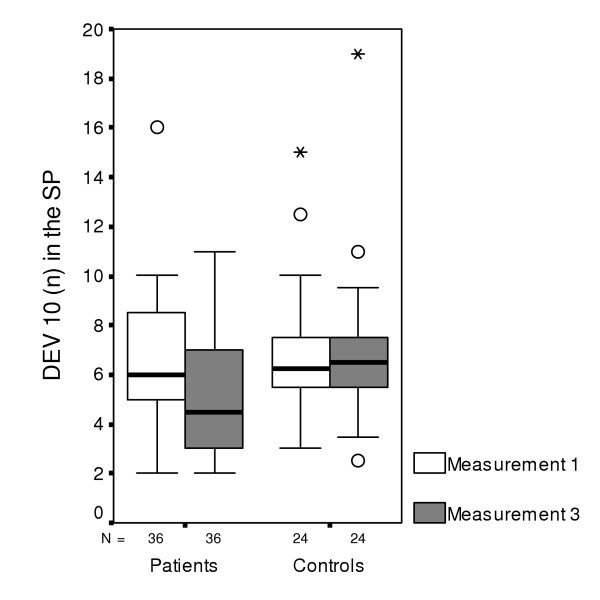
**Post-exercise measurements 1 and 3**. DEV 10 (n) in the sagittal plane (SP) post-exercise measurements 1 and 3, for the injured leg of the patients and the uninjured legs of the control group. The circles denote outliers (i.e., cases with values between 1.5 and 3 box lengths from the upper or lower edge of the box), and the asterisks denote extreme values (i.e., cases with values more than 3 box lengths from the upper or lower edge of the box).

In a previous study [26], we observed decreasing values in the three measurements, indicating a learning effect. In another study [36], fatigue was shown to interfere with this learning process, which is in agreement with the results that we found on the uninjured leg and in the control group. However, the injured leg reacted differently from the uninjured one, and the control group when the first and third of the post-exercise measurements were compared. It was assumed that the first measurement could provide us with the instantaneous value of the ability to maintain postural control in single-limb stance. The results showed that the third measurement was lower, or tended to be lower, than the first in the injured leg, regarding average speed and DEV 10 in both planes. No such effect was, however, found in the uninjured leg or in the control group (Table 3). The inter-group comparisons for these post-exercise measurements showed greater effects in the injured leg than in the control group in average speed in the FP, and a tendency towards greater effects in the other three variables (Table 4 and Figures 7, 8, 9, 10). This finding indicates that balance standing on one leg may be improved during the recovery period, and that a learning process may be needed in the injured leg after exercise. A different strategy in the injured leg than in the uninjured one has been reported in individuals with ACL injury [37]. In that study, Di Fabio et al. [37], found that postural responses, measured with external perturbations while standing on a force platform, could be unilaterally restructured and preprogrammed to compensate for the injury.

Mechanoreceptors in the ACL contribute to the neuromuscular control of the muscle tonus around the knee joint via the reflex arc (i.e., reflex from joint afferents to the muscle spindles via the gamma motoneurons), and therefore to the stabilization of the knee joint [32]. Decreased proprioception [11,12], increased joint laxity in the knee joint [14,38], and a delay in muscle response in leg muscles [13,14] have been described after fatiguing exercise. In these studies, uninjured subjects were tested. The activity of joint receptors, muscle spindles and Golgi tendon organs may be reduced by fatigue, resulting in proprioceptive deficiency in muscle receptors and loss of muscular reflexes responsible for joint stability [10]. Since this afferent information is important for the maintenance of postural control [32], this may lead to decreased muscle response and poorer ability to maintain balance. The increase in joint laxity following fatigue has been suggested to be due to reduced muscle tone [38], viscoelastic changes in the collagenous tissues of the knee and fatigued muscle stabilizers [14], and results in inadequate ligament mechanoreceptor feedback, which is required to elicit the muscular reflexes responsible for joint stability [10]. It has been suggested that muscle receptors are the primary determinant of joint position sense, and capsular receptors may have a secondary role [12,32]. Therefore, the decreased proprioceptive ability following fatigue has been proposed to be due to the decrease in muscle receptor activity [11,12]. Since defects in proprioception [39], impaired postural control [2-5], increased joint laxity [32], and a delay in muscle reaction time [4,40,41] are present already in an unfatigued state in individuals with ACL injury, they may, at least theoretically, be more affected by fatigue than uninjured subjects. Although we found effects of exercise after a short period of cycling above 60% of the predicted HR_max_, it is possible that greater effects of exercise on balance in single-limb stance may be seen after longer durations of exercise than in the present study. It is also possible that larger effects of exercise may be reflected in more challenging measures of postural control, such as dynamic balance tests. Since, to our knowledge, this is the first study on the effects of fatigue on postural control in patients with ACL injury, the clinical relevance of our results remains unclear. More research is needed to further study whether this may be related to an increased risk of further injuries.

The lower average speed and lack of difference in DEV 10 in the patients compared to the control group, indicate sway movements at a lower speed with retained amplitudes to be neuromuscular adaptive strategies, rather than more rapid, smaller adjustments (Table 5). These strategies may be the result of decreased proprioception [39], and a delay in muscle reaction time [4,40,41], which has been reported after an ACL injury, and thus, these strategies may be needed to generate sufficient afferent impulses to obtain dynamic stabilization of the knee joint. Another possible explanation may be that the patients had all undergone neuromuscular training, which may have affected the strategies of maintaining balance in single-limb stance compared with the control group who had not undergone such training. The clinical relevance of the fact that the patients' post-exercise values approached those of the control group, remains, however, unclear. More research is needed to elucidate this further.

## Conclusions

The results of the present study showed no differences in the effects of exercise between the patients and the controls. However, the injured leg was more affected or tended to be more affected directly after exercise than the control group, which indicates that patients with ACL injury react differently regarding their ability to maintain balance in single-limb stance after short-duration, sub-maximal cycling, than a control group of uninjured subjects. The patients used sway movements at a lower speed with retained amplitudes, which may be an expression of neuromuscular adaptive strategies.

## Competing interests

The author(s) declare that they have no competing interests.

## Authors' contributions

EA participated in the design of the study, participated in collecting the data, performed the statistical analysis, and drafted the manuscript. DR participated in collecting the data. EH participated in the progress and revision of the manuscript. TF participated in the design of the study, and in the progress and revision of the manuscript. All authors read and approved the final manuscript.

## Pre-publication history

The pre-publication history for this paper can be accessed here:



## Figures and Tables

**Table 1:** Pre- and post-exercise values for the stabilometric variables.

	**Patients**								**Control group**			
					
	**Injured leg**				**Uninjured leg**							
	
	**Pre-exercise Mean (SD)**	**Post-exercise Mean (SD)**	**Mean diff. ** **(99% CI)**	**P-value**	**Pre-exercise Mean (SD)**	**Post-exercise Mean (SD)**	**Mean diff. ** **(99% CI)**	**P-value**	**Pre-exercise Mean (SD)**	**Post-exercise Mean (SD)**	**Mean diff. ** **(99% CI)**	**P-value**
**Frontal plane**												
Average speed (mm·s^-1^)	20.8 (5.3)	22.6 (5.7)	1.8 (0.6–3.1)	0.0003	21.4 (5.8)	23.2 (5.6)	1.8 (0.7–2.9)	0.0001	23.2 (5.2)	24.4 (5.2)	1.2 (-0.0–2.5)	0.01
DEV 10 (n)	3.5 (2.6)	4.4 (2.7)	0.9 (-0.3–2.0)	0.048	3.4 (2.1)	4.7 (2.6)	1.3 (0.6–2.1)	0.00002	3.5 (1.8)	4.4 (2.3)	0.9 (-0.2–2.0)	0.03
**Sagittal plane**												
Average speed (mm·s^-1^)	20.3 (5.4)	22.8 (6.6)	2.5 (1.1–3.8)	0.00002	20.1 (4.4)	22.6 (4.9)	2.5 (1.1–3.8)	0.00002	24.0 (5.0)	25.2 (6.2)	1.2 (-0.7–3.0)	0.09
DEV 10 (n)	6.0 (3.0)	6.0 (2.8)	0.0 (-1.0–1.1)	0.94	5.4 (2.1)	5.7 (2.2)	0.3 (-0.4–1.1)	0.23	6.5 (2.5)	6.6 (2.5)	0.1 (-0.7–0.9)	0.78

Mean (SD), mean difference (99% CI) (post-exercise minus pre-exercise), and P-value for stabilometric variables in the injured and uninjured legs, and in the control group before (pre-exercise) and after (post-exercise) short-duration sub-maximal cycling. A level of P < 0.01 indicates statistical significance.

**Table 2:** Effects of exercise (post-exercise minus pre-exercise) patients vs. controls.

	**Injured leg vs. control group**		**Uninjured leg vs. control group**	
	
	**Mean diff. (99% CI)**	**P-value**	**Mean diff. (99% CI)**	**P-value**
**Frontal plane**				
Average speed (mm·s^-1^)	0.6 (-1.2–2.4)	0.37	0.6 (-1.0–2.2)	0.33
DEV 10 (n)	-0.03 (-1.6–1.6)	0.95	0.4 (-0.8–1.6)	0.34
**Sagittal plane**				
Average speed (mm·s^-1^)	1.3 (-0.9–3.4)	0.12	1.3 (-0.9–3.5)	0.12
DEV 10 (n)	-0.06 (-1.5–1.4)	0.92	0.2 (-0.8–1.3)	0.54

Mean difference (99% CI) (post-exercise minus pre-exercise), and P-value for stabilometric variables for the injured leg vs. control group, and the uninjured leg vs. control group. A level of P < 0.01 indicates statistical significanc

**Table 3:** First and third post-exercise measurements for the stabilometric variables.

	**Patients**								**Control group**			
					
	**Injured leg**				**Uninjured leg**							
	
	**Meas. 1 Mean (SD)**	**Meas. 3 Mean (SD)**	**Mean diff. (98% CI)**	**P-value**	**Meas. 1 Mean (SD)**	**Meas. 3 Mean (SD)**	**Mean diff. (98% CI)**	**P-value**	**Meas. 1 Mean (SD)**	**Meas. 3 Mean (SD)**	**Mean diff. (98% CI)**	**P-value**
**Frontal plane**												
Average speed (mm·s^-1^)	24.6 (7.6)	21.7 (5.5)	-2.9 (-5.0– -0.9)	0.001	24.8 (6.2)	23.8 (6.6)	-1.0 (-2.7–0.7)	0.15	24.8 (6.2)	24.3 (5.5)	-0.5 (-1.8–0.7)	0.25
DEV 10 (n)	5.7 (3.8)	4.0 (2.5)	-1.7 (-3.2–-0.4)	0.004	5.7 (3.7)	4.5 (3.1)	-1.2 (-2.6–0.3)	0.06	4.8 (2.6)	4.5 (2.7)	-0.3 (-1.2–0.4)	0.24
**Sagittal plane**												
Average speed (mm·s^-1^)	23.8 (6.9)	22.3 (6.5)	-1.5 (-2.9–-0.1)	0.03	23.1 (5.4)	22.9 (5.8)	-0.2 (-1.7–1.3)	0.72	25.3 (6.4)	25.0 (6.2)	-0.3 (-1.3–0.8)	0.52
DEV 10 (n)	6.6 (2.8)	5.6 (3.8)	-1.0 (-2.0–-0.0)	0.02	5.7 (2.3)	6.1 (3.1)	0.4 (-0.9–1.7)	0.47	6.8 (2.7)	6.9 (3.2)	0.1 (-1.1–1.5)	0.75

Mean (SD), mean difference (98% CI) (measurement 3 minus measurement 1), and P-value for the first and third of the post-exercise measurements for the stabilometric variables in the injured and uninjured legs and in the control group. A level of P < 0.02 indicates statistical significance.

**Table 4:** Effects of exercise (measurement 3 minus measurement 1) patients vs. controls.

	**Injured leg vs. control group**		**Uninjured leg vs. control group**	
	
	**Mean diff. (98% CI)**	**P-value**	**Mean diff. (98% CI)**	**P-value**
**Frontal plane**				
Average speed (mm·s^-1^)	-2.4 (-4.7–-0.0)	0.02	-0.5 (-2.4–1.6)	0.62
DEV 10 (n)	-1.4 (-3.0–0.2)	0.04	-0.8 (-2.4–0.8)	0.25
**Sagittal plane**				
Average speed (mm·s^-1^)	-1.2 (-2.9–0.6)	0.13	0.05 (-1.8–1.8)	0.95
DEV 10 (n)	-1.1 (-2.7–0.4)	0.08	0.2 (-1.6–2.1)	0.78

Mean difference (98% CI) (measurement 3 minus measurement 1), and P-value for stabilometric variables the injured leg vs. control group, and the uninjured leg vs. control group. A level of P < 0.02 indicates statistical significance.

**Table 5:** Patients vs. control group.

	**Pre-exercise**					**Post-exercise**				
	
	**Injured leg**		**Control group**		**Uninjured leg**	**Injured leg**		**Control group**		**Uninjured leg**
	**Mean** **(SD)**	**P-value** (inj vs. control)	**Mean** **(SD)**	**P-value** (uninj vs. control)	**Mean** **(SD)**	**Mean** **(SD)**	**P-value** (inj vs. control)	**Mean** **(SD)**	**P-value** (uninj vs. control)	**Mean** **(SD)**

**Frontal plane**										
Average speed (mm·s^-1^)	20.8 (5.3)	0.09	23.2 (5.2)	0.23	21.4 (5.8)	22.6 (5.7)	0.21	24.4 (5.2)	0.41	23.2 (5.6)
DEV 10 (n)	3.5 (2.6)	0.97	3.5 (1.8)	0.87	3.4 (2.1)	4.4 (2.7)	0.98	4.4 (2.3)	0.60	4.7 (2.6)
**Sagittal plane**										
Average speed (mm·s^-1^)	20.3 (5.4)	0.009	24.0 (5.0)	0.002	20.1 (4.4)	22.8 (6.6)	0.16	25.2 (6.2)	0.07	22.6 (4.9)
DEV 10 (n)	6.0 (3.0)	0.51	6.5 (2.5)	0.06	5.4 (2.1)	6.0 (2.8)	0.44	6.6 (2.5)	0.15	5.7 (2.2)

Mean (SD) pre-exercise and post-exercise for stabilometric variables in the injured (inj) and uninjured (uninj) legs, and in the control group, and P-value for the injured leg vs. control group, and the uninjured leg vs. control group. A level of P < 0.01 indicates statistical significance.
